# Evidence of extensive non-allelic gene conversion among LTR elements in the human genome

**DOI:** 10.1038/srep28710

**Published:** 2016-06-27

**Authors:** Beniamino Trombetta, Gloria Fantini, Eugenia D’Atanasio, Daniele Sellitto, Fulvio Cruciani

**Affiliations:** 1 Dipartimento di Biologia e Biotecnologie “Charles Darwin”, Sapienza Università di Roma, Rome, Italy; 2 Istituto di Biologia e Patologia Molecolari, CNR, Rome, Italy; 3 Istituto Pasteur-Fondazione Cenci Bolognetti, Sapienza Università di Roma, Rome, Italy

## Abstract

Long Terminal Repeats (LTRs) are nearly identical DNA sequences found at either end of Human Endogenous Retroviruses (HERVs). The high sequence similarity that exists among different LTRs suggests they could be substrate of ectopic gene conversion events. To understand the extent to which gene conversion occurs and to gain new insights into the evolutionary history of these elements in humans, we performed an intra-species phylogenetic study of 52 LTRs on different unrelated Y chromosomes. From this analysis, we obtained direct evidence that demonstrates the occurrence of ectopic gene conversion in several LTRs, with donor sequences located on both sex chromosomes and autosomes. We also found that some of these elements are characterized by an extremely high density of polymorphisms, showing one of the highest nucleotide diversities in the human genome, as well as a complex patchwork of sequences derived from different LTRs. Finally, we highlighted the limits of current short-read NGS studies in the analysis of genetic diversity of the LTRs in the human genome. In conclusion, our comparative re-sequencing analysis revealed that ectopic gene conversion is a common event in the evolution of LTR elements, suggesting complex genetic links among LTRs from different chromosomes.

Human Endogenous Retroviruses (HERVs) are inherited DNA proviruses arising from retroviral infections of germ-line cells and subsequent integration into the host genome^1^,^2^.

After integration, the provirus may experience numerous amplifications. The human genome is estimated to contain thousands of copies of these interspersed repetitive elements. In fact, they make up a significant fraction (~8%) of its sequence content^3^,^4^,^5^. Many families of HERVs exhibit high transcriptional activity in different human tissues, and both beneficial^6^,^7^,^8^ and detrimental effects^8^,^9^,^10^ have been described. Similarly to other endogenous proviruses, they consist of a 5–10 kb sequence which code for viral proteins, flanked by two Long Terminal Repeats (LTRs) (0.3–1.6 kb in length) that contain regulatory elements for viral protein expression^11^.

Although the majority of HERV genes are highly defective due to several inactivating mutations in their coding sequences (ranging from single nucleotide changes to large deletions), LTR elements still retain their regulatory activity participating in the regulation of cell-type specific gene expression in mammals^12^,^13^,^14^,^15^. Furthermore, LTRs flanking a HERV might recombine with each other leading to the excision of the coding region, thus becoming solitary elements (“Solo LTRs”)^16^,^17^. Solo LTRs are quite common in the human genome and play important roles in genome evolution^18^,^19^.

In general, it has been hypothesized that LTRs may be important contributors to the genome plasticity of the host species due to their capability to undergo ectopic recombination events such as unequal crossing over^19^.

Recent data suggested that ectopic (non-allelic) gene conversion might have played a role in the evolution of flanking LTRs in humans^20^. Gene conversion mediates the transfer of genetic information from a “donor” sequence to a highly similar “acceptor”^21^ and this can have two major effects: on one hand, it can act as a homogenizing force, increasing similarity between paralogous sequences, while on the other hand, it can generate an excess of genetic diversity among allelic copies of the “acceptor” sequence. Although episodes of non-allelic gene conversion between flanking LTRs have been previously hypothesized through an inter-species comparative analysis in different primates^18^,^19^,^20^, there is little information available regarding the dynamics and the pervasiveness of this process in humans (also considering “solo LTR” elements).

Intra-species sequence diversity comparisons among different individuals could shed light on the dynamics of gene conversion among LTRs. However, in most of the genome, the phenomenon of diploidy complicates the comparative sequencing strategies because of sequence diversity between alleles. The haploid Male Specific region of the human Y chromosome (MSY) has no such limitations. Moreover, because of its genetic features, the MSY is an ideal tool to study the dynamics of ectopic gene conversion in humans^22^,^23^,^24^,^25^.

The purpose of this study is to gain new insights into the evolutionary history of LTRs by determining the nature and the extent at which gene conversion occurs among these elements. To this end, we carried out an intra-species phylogenetic analysis of 52 LTRs belonging to 14 different subfamilies on several human Y chromosomes, representing a wide range of worldwide human MSY diversity.

A recent history of gene conversion among LTRs associated with the MSY may be postulated: 1) by the identification of a higher than expected nucleotide diversity within LTR elements, which act as gene conversion “acceptor” sequences^22^,^24^,^26^ and 2) by the occurrence of multiple phylogenetically equivalent Single Nucleotide Polymorphisms (SNPs) occurring at closely spaced positions (clusters of SNPs) showing the derived allele to be the same as the paralogous base on the donor^23^,^24^,^25^,^27^.

By exploiting the haploid nature of the human Y chromosome and its known phylogeny, we hypothesized that some LTR elements have acted as gene-conversion “acceptors” and re-sequenced two of them in a wider sample set. Our comparative re-sequencing analysis provide direct evidence of new LTR-related gene conversion hotspots on the human Y chromosome and suggests complex genetic links among elements spread across the entire human genome. Moreover, we identify a third form of productive recombination of the human MSY, autosome-to-Y gene conversion. Finally, we found that some LTRs are characterized by an extremely high density of polymorphisms showing one of the highest nucleotide diversities in the human genome, as well as a complex patchwork of sequences derived from different elements.

## Results

### Sequence diversity in LTR elements

To determine whether different LTRs (both “solo” elements and HERV-flanking ones) have been engaged in ectopic gene conversion events during recent human evolution, we studied the genetic diversity of the human Y chromosome in 52 LTRs from 14 different subfamilies (Supplementary Table S1).

Overall, 61 Kb of the MSY were re-sequenced, including LTR elements (about 52 Kb) and their flanking regions (about 9 Kb), in 16 Y chromosomes from different haplogroups of the Y phylogeny.

We found a total of 131 SNPs and 3 indels (V350.2; V418 and V435) of 14, 19 and 120 bp, respectively (Supplementary Table S2). These variants do not include 2 nucleotide positions (28,828,795 in LTR 41 and 24,394,612 in LTR 51) that were invariant in the entire sample set but different from the reference sequence, a finding that can be interpreted as a consequence of reference-specific mutations (in both cases a G to A transition).

The nucleotide diversity for LTR elements was estimated as π = 3.6 ± 0.4 × 10^−4^, which is significantly higher both than that observed for their surrounding regions (π = 2.2 ± 0.4 × 10^−4^) and that previously observed for portions of the X-degenerate region of the MSY (π = 1.5 ± 0.3 × 10^−4^)^24^. This finding suggests that LTR elements might have had a peculiar evolutionary history, which have led to an increase in their genetic diversity.

The 134 polymorphisms here identified did not appear to be equally distributed, with some LTRs showing an extremely high density of SNPs, while others (12/52) were found to be invariant. More specifically, about 26% (35 out of 134) of the variants were found in only two elements, LTR 2 and LTR 24, (27 and 8 polymorphisms, respectively).

**Figure 1:**
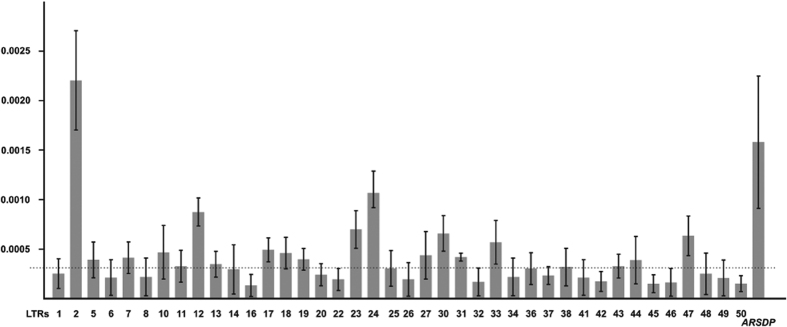
Nucleotide diversity of the LTR elements analysed in the present study. Only LTRs with π > 0 are shown. Dotted line represents the mean value of π. The π value of the *ARSDP* was calculated from data reported in Trombetta *et al*.^24^.

In order to evaluate the relative contribution of each sequenced LTR to the diversity of the global sample, we obtained estimates of the π value by considering all the elements separately (Fig. 1).

The highest nucleotide diversity was observed for LTR 2 (π = 2.2 ± 0.5 × 10^−3^), a value that is about an order of magnitude higher than the average of the X-transposed region and comparable to the estimates obtained for other regions of the Y chromosome in which ectopic gene conversion has already been reported^22^,^24^. In particular, three elements (LTR 2; LTR 12 and LTR 24) showed comparable π values to the most diverse ectopic gene conversion hotspot so far identified on the X-degenerated portion of the MSY (the *ARSDP* hotspot)^24^ (Fig. 1). The high nucleotide diversity here observed among allelic copies could be the consequence of a high mutation rate due to ectopic gene conversion.

### Occurrence of multiple, phylogenetically equivalent and closely spaced SNPs

If gene conversion is operating between LTRs, we would expect to observe a higher number of co-converted sites organized as clustered SNPs (i.e. multiple phylogenetically equivalent SNPs at closely spaced positions) within LTR elements.

**Figure 2:**
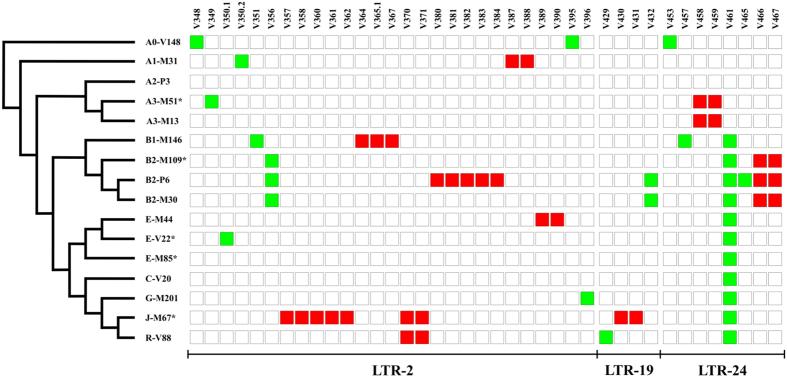
SNPs identified in three LTRs. To the left, a simplified version of the Y-chromosome tree showing the phylogenetic relationships of the 16 sequenced chromosomes. LTRs without clusters of SNPs are not shown. The names of LTRs and SNPs are at the bottom and at the top, respectively. Square colours represent the allelic state for each SNP: White, ancestral allele; red, closely spaced derived SNPs arisen on the same branch of the Y phylogeny; green, «conventionally» derived SNPs.

As expected, the overall pattern of nucleotide diversity was dominated by “conventional” SNPs (i.e. solitary variants on different branches of the Y tree). Nevertheless, we also identified a total of 25 SNPs, grouped into 9 clusters (with each cluster made up of 2 or more mutations), occurring in the same phylogenetic context and at closely spaced positions (less than 50 bp) (Fig. 2). These clusters of SNPs may arise through two possible mechanisms: multiple random mutations or a single ectopic gene conversion event in which a stretch of DNA is replaced by the sequence of a non-identical paralogous region so creating co-converted sites.

Assuming a random distribution of the 134 variants here identified, there is a significant excess (P = 2.3 × 10^−20^) of clustered SNPs that have arisen in the same phylogenetic context. Moreover, the proportion of clustered SNPs (25 out of 134, 19%) was significantly higher (P < 1 × 10^−16^, Fisher’s exact test) than that obtained (about 0.8%) from two recent high-coverage NGS studies of the Y chromosome^28^,^29^ (Table 1).

The nine clusters of SNPs were restricted to 3 LTRs (LTR 2, LTR 19, LTR 24) covering a total of 4.9 Kb (8% of the sequenced region) (Fig. 2). Overall, these findings (a high nucleotide diversity and the presence of clustered SNPs) suggest the involvement of ectopic gene conversion events in shaping the genetic landscape of at least some of the LTR elements here analysed.

To further explore the intensity of ectopic gene conversion in shaping the LTR sequence diversity, we re-sequenced the two elements that showed both the highest diversity and the highest number of clustered SNPs (LTR 2 and LTR 24) in a wider sample set.

**Figure 3:**
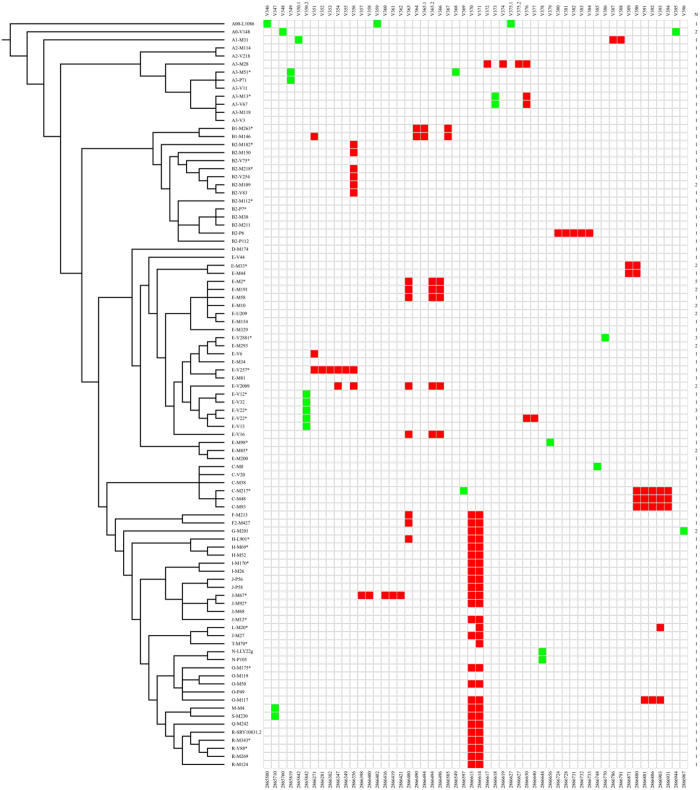
SNPs identified in LTR 2. To the left, a simplified version of the Y-chromosome tree showing the phylogenetic relationships of the Y chromosomes sequenced. The names and position of the SNPs are given at the top and at the bottom, respectively. Square colours represent the allelic state for each SNP: White, ancestral allele; red, closely spaced SNPs arisen on the same branch of the Y phylogeny; green, «conventional» SNPs. N represents the number of Y chromosomes sequenced for each of the haplogroups shown to the left; when N > 1, chromosomes belonging to different subhaplogroups were chosen.

Re-sequencing LTR 2 (1550 bp) in 111 unrelated samples, we found 51 variants, about 70% of which (36/51) were organized in clusters (Fig. 3 and Table 2). We found a total of 13 clusters ranging from 2 bp to 85 bp with a mean length of 23 bp. The longest cluster (cluster 1, 85 bp) was made up of six SNPs found in a single branch of the Y tree (E-V257*) (Fig. 3 and Table 2).

**Figure 4:**
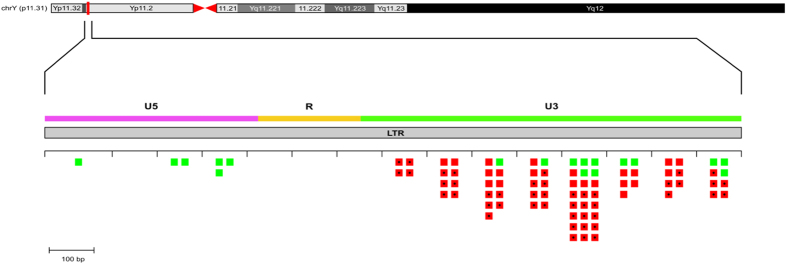
Locations of observed mutational events in the LTR 2 element. Each mutational event is represented by a square placed in the correct 100 bp segment of the element. Green square: conventional SNPs. Red square: clustered SNPs. Square with the dot represents recurrent mutational events. The boundaries of the functional domains of the element are approximate.

Our analysis highlighted that this region was particularly rich in homoplasic variants. Indeed, we found that 14 SNPs (27%) showed evidence of recurrent mutations. The proportion of recurrent events is significantly higher (P < 1 × 10^−4^, Fisher’s exact test) than that reported in Scozzari *et al*. (four out of 2386 positions, 0.2%)^30^ and in Wei *et al*. (172 out of 5865 mutations, 2.9%)^31^. Moreover, each recurrent variant was located within one or more of the thirteen clusters. We counted a total of 34 recurrent mutations, with a single variant (V370) which back-mutated six times on different branches (J-M68, L-M20*, T-M70*, N, O-M119 and O-P49) of the phylogeny (Fig. 3). Interestingly, a single cluster (cluster 3), which arose in three different branches of the phylogeny, (E-M2; E-V2009; E-V16) was entirely made up of homoplasic variants. By exploiting the Y phylogeny and taking into account both recurrent and non-recurrent mutations, we counted a total of 85 putative mutational events. Interestingly, the mutational events appeared to be unevenly distributed, with clustered SNPs and recurrent mutations mainly found in the regulatory region (U3) of the element (Fig. 4).

**Figure 5:**
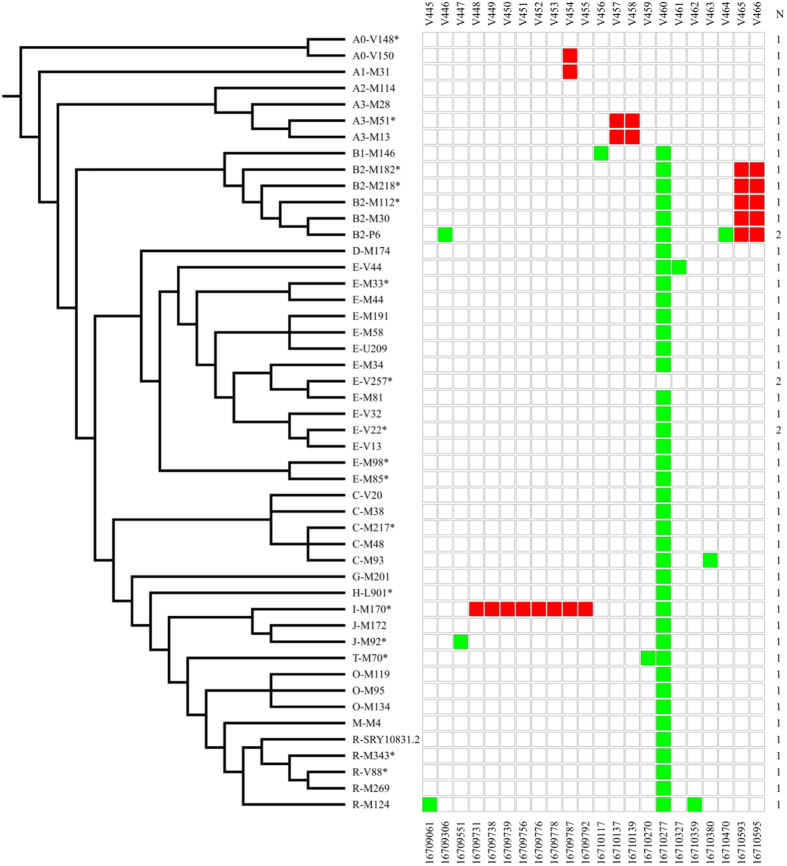
SNPs identified in LTR 24. To the left, a simplified version of the Y-chromosome tree showing the phylogenetic relationships of the Y chromosomes sequenced. The names and positions of the nucleotides are given at the top and at the bottom, respectively. Square colours represent the allelic state for each SNP: White, ancestral allele; red, closely spaced SNPs arisen on the same branch of the Y phylogeny; green, «conventional» SNPs. N represents the number of Y chromosomes sequenced for each of the haplogroups shown to the left; when N > 1, chromosomes belonging to different subhaplogroups were chosen.

The re-sequencing of LTR 24 (1828 bp) in 51 unrelated samples showed a similar although less extreme picture. We found a total of 23 SNPs (Fig. 5 and Supplementary Table S2), about 56% of which (13 out of 23) were in three clusters (Fig. 5 and Table 2). One of these (cluster 14) is composed of eight SNPs and it covers a total of 63 bp in the I-M170* haplogroup. About 8.6% (2 out of 23) of the variants was recurrent: one SNP (V460) back-mutated in the E-V257* branch and the other (V454) recurred in three different branches of the phylogeny (A0-V150, A1-M31 and I-M170*).

In conclusion, the high number of clustered SNPs here observed cannot be simply explained by random mutations. In principle, the unusual mutational patterns here observed could be explained by double crossovers between paralogs with reciprocal exchange of slightly different segments of DNA. However, due to the short length of the observed sequence changes, the most parsimonious explanation remains ectopic gene conversion^21^.

### Donor sequences involved in ectopic gene conversion

Assuming that a cluster is due to ectopic gene conversion, it should be possible to identify donor sequences within the genome. Through whole genome alignments of gene-converted tracts, we identified different donor sequences involved in the observed gene conversion events. In order to minimize the probability of observing a donor sequence by chance, we only analysed clusters of SNPs with length ≥ 3bp, thus excluding cluster 12, which contains only two contiguous SNPs (V370 and V371). A genomic region was considered a putative “donor” if it shows 100% sequence identity with the derived state of each cluster. For about 67% of the clusters (10 out of 15), we were able to find univocally one single donor, whereas for five clusters, multiple putative donors were identified (Table 2).

The minimum observed tract length ranged from 3 bp to 86 bp, while the maximum gene-conversion tract, measured as the distance between the two nearest non-converted Paralogous Sequence Variants (PSVs) flanking the converted site, ranged from 14 to 450 bp, with an average length of 99 bp. In total, at least 14 different LTRs acted as donor sequences in gene conversion events involving LTR 2 and LTR 24 as acceptors. We observed both intra- and inter-chromosomal gene conversion events, and in at least two cases (cluster 4 and 9), the single donor sequence was located on an autosome (Table 2), which suggests that the MSY is able to recombine, in the form of gene conversion, not only with the X chromosome^23^,^24^,^25^,^27^,^32^ but also with autosomes. Interestingly, the wide cluster 14 was generated by intra-LTR conversion, in which the transfer of genetic information occurred between two duplicated regions within the same elements (LTR 24). In turn, clusters 15 and 16 were generated by gene conversion between two HERV-flanking elements: LTR 24 (acceptor sequence) and LTR23 (donor sequence).

In total, we found evidence for three kinds of gene conversion (GC), based on the nature of the donor/s sequence: (1) multiple-donors GC (LTR 2), where different LTR elements from the Y chromosome or autosomes acted as donor sequences; (2) single-donor GC between HERV-flanking LTRs (LTR 24) and (3) intra-LTR GC (LTR24), where ectopic gene conversion occurred between duplicated elements within a single LTR.

## Discussion

One of the most remarkable features of the human genome is that about one half of it is composed of interspersed repetitive elements. A part of these elements are remnants of ancestral retroviruses, called Human Endogenous Retroviruses (HERVs), which originated through successive waves of ancient infection of germ cells and subsequent incorporation into the host’s genome. Following integration, the provirus will be parasitically maintained within the genome and will be inherited vertically form parent to offspring in a Mendelian fashion. The inserted provirus is an identical DNA copy of the RNA viral genome with the exception that it is flanked by two identical Long Terminal Repeats (LTRs) containing transcriptional regulatory elements such as enhancers and promoters. These repeats play important roles in viral gene expression and, although most proviral genes are inactivated by several mutations, LTRs could preserve their regulative functions, thereby imposing a regulatory activity upon host cell genes^33^,^34^. Apart from affecting gene expression, LTRs could significantly influence genome evolution. Indeed, they can be involved in ectopic crossing over, generating structural changes within the host genome. A major effect of crossing over between LTRs flanking a HERV is the excision of the viral genes and the establishment of a single element named solo-LTR.

By comparative inter-species analysis, it has been proposed that gene conversion among LTRs could have played a role in the sequence evolution of these elements^18^,^19^,^20^, but no direct evidence of this has been reported in humans. In the present study, by exploiting the haploid nature of the MSY, we demonstrated the existence of several non allelic gene conversion events among LTRs, highlighting that this molecular process can be highly effective in modulating the sequence landscape of these elements on the human Y chromosome. More specifically, using an intra-species phylogenetic study, we defined at least two LTRs (LTR 2 and LTR 24) as two new gene conversion hotspots characterized by an extremely high density of SNPs, as well as a complex patchwork of sequences derived from different LTRs spread across the entire genome.

The nucleotide diversity of the LTRs here analysed was found to be higher than that calculated for their surrounding regions. In particular, LTR 2 showed one of the highest nucleotide diversities (π = 2.2 ± 0.5 × 10^−3^) of the human genome. Excluding the HLA locus^35^, few genomic regions have been reported to show similar diversity values. Examples include the autosomic LHB/CGB loci (shaped by ectopic gene conversion)^26^, the X-to-Y gene conversion hotspots found within the MSY^24^ and a few other regions where gene conversion has been previously identified^22^,^36^. The high values of diversity here observed provide a clear picture of the remarkable ability of gene conversion to increase diversity among allelic sequences which act as acceptor elements.

Our analysis was mainly based on the identification of clusters of SNPs (i.e. groups of two or more SNPs occurring in close proximity and on the same branch of the Y phylogeny, thus indicating that they probably arise at the same time as a single event). Regarding Y chromosome phylogeny, assuming no gene conversion and random mutations, one would expect to observe different SNPs both randomly distributed along the Y chromosome tree and physically distanced from each other. This is the situation observed in a recent high coverage NGS study of the Y chromosome^28^, where the analysis of several Mb in hundreds of Y chromosomes led to the identification of a very low frequency (0.8%) of clustered SNPs (Table 1). The opposite was observed for two of the LTR elements here analysed (LTR 2 and LTR 24) where 70% and 56% (respectively) of clustered SNPs were found. The most parsimonious explanation for this observation is that recent gene conversion events have played a role in shaping the genetic diversity of these elements. Indeed, if there are single nucleotide differences (that are physically close to one another) between the “donor” and the “acceptor” sequence, the main effect of the replacement of a stretch of DNA by a paralogous sequence (gene conversion) is to create simultaneously new polymorphisms close to each other and in the same phylogenetic context (clusters of SNPs)^23^,^24^,^25^. The uneven distribution of clusters among different elements suggests that the LTRs here analysed could, as acceptor sequences of gene conversion, act differently with some elements being probably more frequently involved.

Gene conversion, together with the primary effect of generating clusters of SNPs, may generate homoplasies (in the form of recurrent mutations, back mutations or SNPs with more than two alleles) along the Y phylogeny^24^,^32^,^37^. LTR 2 and LTR 24 were found to be significantly enriched with homoplasic variants compared to other regions of the Y chromosome. The observed excess of recurrent mutations within gene conversion hotspots raises important issues about the use of SNPs within the LTRs as stable markers in phylogenetic tree reconstruction and their potential use in forensic applications such as Ancestry Informative Markers^32^,^37^,^38^,^39^. The use of these SNPs as stable variants could erroneously alter the structure of the tree, obscuring phylogenetic signals from other markers and making it difficult to understand the evolutionary relationships among different Y chromosomes. The identification of abundant gene conversion events among LTRs on the human Y chromosome means that care is needed when using these regions in phylogenetic studies and that all the SNPs that may have been arisen by gene conversion should be excluded from evolutionary analysis.

Ectopic gene conversion always initiates with a double strand break (DSB) which represents one of the most deleterious forms of damage of genetic material as it can lead to genome instability and cell death^40^. DSBs are strong inducers of Homologous Recombination (HR), which will potentially lead to chromosomal aberrations if the template sequence is a paralogous region rather than an allelic one^41^. However, 98% of the DSBs, which are repaired by HR, is resolved by gene conversion rather than crossing over^21^. Recently, Trombetta *et al*.^25^ highlighted an association, on the human Y chromosome, between gene conversion hotspots and regions of genomic instability that show a high frequency of DSBs^25^. In this view, it is tempting to speculate that differences in the occurrence of gene conversion among the LTRs here analysed may reflect a difference in the frequency of DSB formation and that the two elements (LTR 2 and LTR 24) showing the strongest conversion signals could be the most fragile elements.

Through whole genome alignments of gene-converted tracts, we identified the donor sequences involved in the observed gene conversion events. However, it was not possible to determine a single donor sequence for all the events. Differently from other previously identified Y chromosome gene conversion hotspots^24^,^25^,^27^, the LTR 2 region was found to be converted by multiple donor sequences. Intra-chromosomal Y-to-Y gene conversion was much more represented than inter-chromosomal events, probably due to the linkage between donor and acceptor sequences. Indeed, it is known that the physical distance between acceptor and donor sequences is a factor affecting the rate of gene conversion. More precisely, the frequency of gene conversion is inversely proportional to the distance between the interacting sequences in *cis*^42^, and a higher rate of conversion has been observed between linked as opposed to unlinked paralogs in mice^43^. In this view, since the two new hotspots here identified have potentially thousands of donors, the DSBs are probably mainly repaired by the potential donor sequences that are linked to them on the same chromosome. DSBs may also induce inter- and intra-chromosomal Ectopic Recombination (ER) generating several chromosomal rearrangements (such as transposition, inversion or deletions) that may have a dramatic impact on the organization of the genome structure. Little is known about the quantitative relationship between gene conversion rate and ectopic recombination rate on the same *locus*, but the identification of abundant gene conversion on some LTRs leads to speculate that these elements can also be ER hotspots, therefore particularly important regions for a hypothetical structural re-organization of the human genome.

To date, considering the location of the donor sequences, only two forms of non allelic gene conversion have been found to be active on the MSY: The Y-to-Y gene conversion^22^,^44^,^45^ and the X-to-Y one^23^,^24^,^25^,^27^,^32^,^46^. In this study, for the first time, through the identification of donor sequences located on different autosomal chromosomes, we identified a third form of recombination for the MSY: the autosome-to-Y gene conversion. Thus, we further complicate the evolutionary picture of the MSY by demonstrating that the sequence landscape of this genomic region could also be modulated by autosomal sequences.

LTR 2 is a solo-LTR in which we identified 13 different clusters that originated by gene conversion with at least 12 different donors spread across the entire genome. The high number of clusters within LTR 2 does not necessarily reflect a more intense gene conversion activity, but it could be the consequence of the involvement of a large number of different sequences acting as donors. The presence of different LTRs acting as donors could be a continuous source of variants and it can greatly increase the allelic diversity of the acceptor. This is mainly because the donor sequences are free to mutate and accumulate single nucleotide differences compared to the acceptor so that the dynamic balance between mutation and gene conversion will never lead to a complete sequence identity among the interacting sequences. The main effect of the presence of many different donors is that LTR 2 was found to be a sequence patchwork of short segments of DNA derived from different elements. Conversely, when only one donor is involved (as in the case of X-to-Y conversion hotspots described in^23^ and in^25^), gene conversion events are expected to decrease the diversity between donor and acceptor sequences. As a consequence, a conversion-mutation dynamic equilibrium will be reached and the similarity between interacting sequences will increase and arrive at a point in which conversion events will no longer be detectable due to the lack of differences between donor and acceptor.

LTRs are composed of three separate domains: U3, R and U5. Interestingly, within LTR 2 the SNPs appeared to be unevenly distributed, and all the clustered SNPs generated by gene conversion were located within the U3 portion of the element (Fig. 4). The U3 region contains several binding sites for different transcription factors with regulatory activities that tend to be lost by the cell, through a gradual accumulation of mutations^16^,^47^,^48^. At the moment, it is not possible to determine why there is a preferential accumulation of mutations in this domain, but we cannot rule out a role for gene conversion in maintaining or silencing the functional activity of this regulatory region.

Exploring in the chimpanzee genome the distribution of the human LTRs here analysed, we noted that in the orthologous region of the flanking-HERV elements LTR 23 and LTR 24, only one solo-LTR is present, which probably originated through a recombination event between the two elements. By aligning the human LTRs (LTR 23 and LTR 24) with the solo-LTR in the chimpanzee, we mapped the break point of the crossing-over event leading to the formation of the solo-LTR in the simian lineage. We noticed that the break point perfectly overlaps with cluster 16, suggesting that this region was a non-allelic recombination hotspot in both lineages. The existence of a non-allelic recombination hotspot shared between two different species suggests that it pre-dates the human-chimp speciation. This finding adds to previous evidence in favour of a greater conservation in time of non-allelic recombination hotspots (NAHR) compared to allelic recombination hotspots (AHR). Many AHR hotspots that have been described for the human genome have no counterpart in the chimpanzee^49^,^50^ indicating a fast turn-over of these elements, whereas recent studies have shown that NAHR are often shared between evolutionarily distant species of primates^22^,^24^,^25^,^44^,^50^.

The identification of gene conversion among LTRs casts some doubts on the use of Next Generation Sequencing (NGS) to identify new polymorphisms within these elements. Indeed, NGS is based on the alignment to the reference genome of very short sequences (50–200 bp) called reads. Since the length of the gene conversion tracts here identified is comparable with the length of the reads generated by NGS, it is possible that a “converted” sequence can be confused with the donor sequence and wrongly aligned to the paralogous region. Alternatively, the reads could be discarded by the alignment processes or deep-sequencing-associated bioinformatics analyses may consider closely spaced SNPs (a common signal in gene conversion) as false positives and discard them. As a consequence, detecting true SNPs in gene-converted repetitive elements may be difficult when using current short-read next generation sequencing techniques and the final result may consist in an underestimation of genetic variability in duplicated regions subjected to non-allelic gene conversion. To assess this topic, we explored the pattern of nucleotide diversity within LTR 2 from a recent paper in which 456 complete Y chromosomes belonging to different branches of the Y phylogeny were re-sequenced using NGS methodologies^28^. We noticed that regarding LTR 2, Karmin *et al*.^28^ identified only 7 true SNPs, about 13% of the number we identified (51) by analysing less than a quarter of the samples (Supplementary Table 3). Interestingly, 15 variants identified by Karmin *et al*.^28^ were discarded during the filtering phases. The observed discrepancy could be partially explained by the fact that some of the haplogroups we analysed were not included in the study by Karmin *et al*.^28^, but probably, it is largely due to problems during the alignment and/or the filtering of the SNPs due to the confounding effect of gene conversion. This observation suggests that the real impact of non-allelic gene conversion on the mutability of the genome may be underestimated during NGS studies principally because of the inadequacy of instrumental and/or bioinformatics technologies currently in use. The problem regarding the identification of SNP clusters by NGS analysis can be resolved through the introduction of deep sequencing based on “long reads” of a few thousand bases.

To sum up, our results revise and expand previous research on LTR gene conversion in humans, clearly indicating a strong recent history of gene conversion among different elements and supporting the idea that the sequence landscape of MSY could be modulated by the transfer of genetic information from different genomic loci.

## Material and Methods

### DNA Samples

DNA samples came from the collections of the authors and human Y chromosomes were selected on the basis of their haplogroups, which had been determined in previous studies^29^,^51^,^52^,^53^,^54^,^55^,^56^. Haplogroups selected for the analysis were chosen in order to maximize the coverage of the Y phylogeny. In most cases, DNA was prepared from fresh venous blood. The study was prospectively examined and approved by the “Policlinico Umberto I, Sapienza Università di Roma” ethical committee (document number 1158/13). The experiments were carried in accordance with the guidelines approved for research on human samples and informed consent was obtained from all participants.

### Identification of SNPs on the Human Y Chromosome

Overall, 61 kb from the MSY was sequenced for the first sample set (16 samples), whereas a total of 111 samples and 51 samples were sequenced for LTR 2 and LTR 24, respectively. We designed PCR and sequence primers (available on request) for each LTR on the basis of the Y chromosome sequence reported in the February 2009 assembly of the UCSC Genome Browser using Primer3 software (http://genome.ucsc.edu/; http://frodo.wi.mit.edu/primer3/). Sequencing templates were obtained through PCR in a 50 ml reaction containing 50 ng of genomic DNA, 200 mM of each deoxyribonucleotide, 2.5 mM MgCl_2_, 1 unit of Taq polymerase, and 10 pmol of each primer. A touch-down PCR program was used with an annealing temperature decreasing from 62 to 55 C over 14 cycles, followed by 30 cycles with an annealing temperature of 55 C. Following DNA amplification, the PCR products were purified using the 715QIAquick PCR purification kit (Qiagen, Hilden, Germany). Cycle sequencing was performed using the BigDye Terminator Cycle Sequencing Kit with Amplitaq DNA polymerase (Applied Biosystems, Foster City, CA) and an internal or PCR primer. Cycle sequencing products were purified by ethanol precipitation and run on an ABI Prism 3730XL DNA sequencer (Applied Biosystems). Chromatograms were aligned and analysed for mutations using Sequencher 4.8 (Gene Codes Corporation, Ann Arbor, MI).

### Data Analysis

Gene conversion between LTRs can have two effects: On the one hand, it can increase their overall sequence similarity; on the other hand, when it involves single nucleotide differences between donor and acceptor sequences, it can generate an excess of genetic diversity among allelic copies^24^. This is because the paralogous bases on the donor sequence will change the bases on the acceptor chromosome^24^,^24^ and new Y chromosome SNPs will be observed in the population. If gene conversion events among LTRs contributes to the variation of these elements, we expect to find an excess of multiple derived variants which are both physically neighbouring and phylogenetically equivalent mutations (cluster of SNPs), where the Y-linked derived alleles are the same as the paralogous bases on the donor. The success of this analysis depends on the correct inference concerning the direction of the mutation and the identification of an ancestral donor sequence. The direction of the mutation for each Y-linked polymorphism was unambiguously determined by placing it in the context of the well-known intra-specific human Y chromosome phylogeny^28^,^29^,^30^,^55^. The P value for the observed number of clustered SNPs was obtained by using a random permutation test. More specifically, we randomized the distribution for the 136 variants here identified within both the 61,165 bp sequenced and the 30 branches of the Y chromosome tree built with the 16 chromosomes here analyzed (Fig. 2) (using the “rand between” function of Microsoft Excel software). We then counted the number of times we obtained two or more SNPs within a distance of no more than 50 bp and in the same phylogenetic context (i.e. on the same branch of the tree). This process was replicated 1,000 times, which produced the null distribution of clusters of SNPs. We determined the P value of observing 25 out of 134 SNPs in clusters assuming that their null distribution follows a Poisson distribution.

A cluster of SNPs is defined through the distance between the two outermost variants with the distance between each polymorphism being no more than 50bp.

Nucleotide diversity (p) and its standard deviation (SD)were calculated according to^57^.

Donor sequences were identified by the alignment of the derived state of each cluster against the reference sequence of the human genome (hg19/GRCH 37) using the BLAT function of the UCSC Genome Browser. In most cases, we found donors of the entire cluster length while in other cases, the cluster could be generated by different gene conversion events or by gene conversion and mutation (Table 2). Moreover, in some cases, we found shared SNPs among acceptors and donors (Table 2).

## Additional Information

**How to cite this article**: Trombetta, B. *et al*. Evidence of extensive non-allelic gene conversion among LTR elements in the human genome. *Sci. Rep*. **6**, 28710; doi: 10.1038/srep28710 (2016).

## Supplementary Material

Supplementary Information

## Figures and Tables

**Table 1:** Proportion of clustered SNPs found in different resequencing studies.

Genomic regions	Present study	Karmin *et al*.^28^ (all SNPs)	Karmin *et al*.^28^ (filtered SNPs)	Trombetta *et al*.^28^
	LTRs	Whole MSY	Whole MSY	Unique sequences of MSY (1.5 Mb)
Clustered SNPs	25	350	256	28
Conventional SNPs	109	42035	33072	3362
Total	134	42385	33328	3390
% of clustered SNPs	18.65	0.82	0.77	0.82
P-values (Fisher’s Exact Test, Two Sided) Present study vs…		P < 1 × 10^−16^	P < 1 × 10^−16^	P < 1 × 10^−16^

**Table 2:** Clusters of SNPs identified in present study.

Cluster	LTR	SNPs involved	Total number of SNPs involved	Haplogroup	Position	Length of the cluster (bp)	Donor	Gene conversion tract lengths		Notes
								MIN	MAX	
1	LTR-2	from V351 to V356	6	E-V257*	chrY:2866271–2866356	86	chrY:3966976–3968339	86	246	V355 shared SNP with donor (rs371261324)
2	LTR-2	V354, V356	2	E-V12*	chrY:2866347–2866356	10	chrY:3966976–3968339 chrX:8419802–8421096 chr7:145393687–145395025	10	57	
3	LTR-2	V363, V365.2, V366	3	Homoplasic	chrY:2866480–2866496	17	chrY:8166477–8168142	3	14	V363 could be generated by mutation or by a GC event (min 1, max 14)
4	LTR-2	V364, V365.1, V367	3	B1	chrY:2866490–2866505	16	chr10:19239555–19240773	16	69	
5	LTR-2	V372, V374, V375.2, V376	4	A3-M28	chrY:2866617–2866630	14	chrY:7489271–7490822 chrY:28328893–28330444 chrY:24854475–24856026 chrY:25632458–25634009	14	41	Same donors as cluster 8
6	LTR-2	V387, V388	2	A1-M31	chry:2866786–2866791	6	several putative donors	N.A.	N.A.	
7	LTR-2	from V380 to V384	5	B2-P6	chrY:2866724–2866733	10	chrY:6533820–6535364	10	89	
8	LTR-2	V389, V390	2	E-M33	chrY:2866871–2866880	10	chrY:7489271–7490822 chrY:28328893–28330444 chrY:24854475–24856026 chrY:25632458–25634009	10	74	Same donors as cluster 5
9	LTR-2	V376, V377	2	E-V22*	chrY:2866630–2866640	11	chr6:8665762–8667845	11	54	
10	LTR-2	from V390 to V394	5	C-M217	chrY:2866880–2866931	52	chrY:3966976–3968339	24	450	V394 could be generated by mutation or by a GC event from other donors
11	LTR-2	from V391 to V393	3	O-M117	chrY:2866881–2866903	23	chrY:3966976–3968339 chrX:89776222–89776267	23	34	
12	LTR-2	V370, V371	2	Homoplasic	chrY:2866613–2866613	2	N.A.^a^	N.A.^a^	N.A.^a^	
13	LTR-2	V357, V358, V360, V361, V362	5	J-M67*	chrY:2866398–2866421	24	chrY:8166477–8168142	24	83	
14	LTR-24	from V448 to V455	9	I-M170*	chrY:16709731–16709794	63	chrY:16708816–16710643	63	96	INTRA-LTR GC
15	LTR-24	V458, V459	2	A3-M51	chrY:16710137–16710139	3	chrY:16700564–16702229	3	26	single-donor GC between HERV-flanking LTRs
16	LTR-24	V465, V466	2	B2-M182	chrY:16710593–16710595	3	chrY:16700564–16702229	3	49	single-donor GC between HERV-flanking LTRs

^a^Not Analysed. Only clusters >2 bp were analysed.
